# Plasma Lipidomic Patterns in Patients with Symptomatic Coronary Microvascular Dysfunction

**DOI:** 10.3390/metabo11100648

**Published:** 2021-09-22

**Authors:** Jonathan R. Lindner, Brian P. Davidson, Zifeng Song, Claudia S. Maier, Jessica Minnier, Jan Frederick Stevens, Maros Ferencik, Sahar Taqui, J. Todd Belcik, Federico Moccetti, Michael Layoun, Paul Spellman, Mitchell S. Turker, Hagai Tavori, Sergio Fazio, Jacob Raber, Gerd Bobe

**Affiliations:** 1 Knight Cardiovascular Institute, Oregon Health & Science University, Portland, OR 97239, USA; davidsbr@ohsu.edu (B.P.D.); ferencik@ohsu.edu (M.F.); taqui@ohsu.edu (S.T.); belcikt@ohsu.edu (J.T.B.); federico.moccetti@gmail.com (F.M.); layoun@ohsu.edu (M.L.); TAvori@ohsu.edu (H.T.); fazio@ohsu.edu (S.F.); 2 Division of Cardiometabolic Health, Oregon National Primate Research Center, Oregon Health & Science University, Beaverton, OR 97006, USA; 3 Department of Chemistry, Oregon State University, Corvallis, OR 97331, USA; Zifeng.Song@Oregonstate.edu (Z.S.); claudia.Maier@oregonstate.edu (C.S.M.); 4 Linus Pauling Institute, Oregon State University, Corvallis, OR 97331, USA; fred.stevens@oregonstate.edu (J.F.S.); gerd.bobe@oregonstate.edu (G.B.); 5 School of Public Health, Oregon Health & Science University, Portland, OR 97239, USA; minnier@ohsu.edu; 6 Department of Pharmaceutical Sciences of the College of Pharmacy, Oregon State University, Corvallis, OR 97331, USA; 7 Department of Molecular and Medical Genetics, Oregon Health & Science University, Portland, OR 97221, USA; spellmap@ohsu.edu; 8 Oregon Institute of Occupational Health Sciences, Oregon Health & Science University, Portland, OR 97221, USA; Turkerm@ohsu.edu; 9 Department of Behavioral Neuroscience and Neurology, Oregon Health & Science University, Portland, OR 97221, USA; raberjake@gmail.com; 10 Department of Radiation Medicine, Oregon Health & Science University, Portland, OR 97221, USA; 11 Department of Animal and Rangeland Sciences, Oregon State University, Corvallis, OR 97331, USA

**Keywords:** coronary artery disease, lipidomics, microvascular dysfunction, myocardial contrast echocardiography, myocardial ischemia

## Abstract

Coronary microvascular dysfunction (MVD) is a syndrome of abnormal regulation of vascular tone, particularly during increased metabolic demand. While there are several risk factors for MVD, some of which are similar to those for coronary artery disease (CAD), the cause of MVD is not understood. We hypothesized that MVD in symptomatic non-elderly subjects would be characterized by specific lipidomic profiles. Subjects (*n* = 20) aged 35–60 years and referred for computed tomography coronary angiography (CTA) for chest pain but who lacked obstructive CAD (>50% stenosis), underwent quantitative regadenoson stress-rest myocardial contrast echocardiography (MCE) perfusion imaging for MVD assessment. The presence of MVD defined by kinetic analysis of MCE data was correlated with lipidomic profiles in plasma measured by liquid chromatography and high-resolution mass spectrometry. Nine of twenty subjects had evidence of MVD, defined by reduced hyperemic perfusion versus other subjects (beta-value 1.62 ± 0.44 vs. 2.63 ± 0.99 s^−1^, *p* = 0.009). Neither the presence of high-risk but non-obstructive CAD on CTA, nor CAD risk factors were different for those with versus without MVD. Lipidomic analysis revealed that patients with MVD had lower concentrations of long-carbon chain triacylglycerols and diacylglycerols, and higher concentrations of short-chain triacylglycerols. The diacylglycerol containing stearic and linoleic acid classified all participants correctly. We conclude that specific lipidomic plasma profiles occur in MVD involving saturated long-chain fatty acid-containing acylglycerols that are distinctly different from those in non-obstructive CAD. These patterns could be used to better characterize the pathobiology and potential treatments for this condition.

## 1. Introduction

The left ventricular myocardium is in a constant state of high metabolic demand. Yet, the working myocyte contains only a small amount of high-energy phosphate reserves [1]. Accordingly, adequate tissue perfusion is essential for the constant delivery of oxygen and high-energy fuels including fatty acids (FA), glucose, amino acids and ketones; and oxygen for high-efficiency oxidative generation of myocellular adenosine triphosphate (ATP) [2]. Myocardial perfusion can be compromised by a spectrum of diseases, including obstructive coronary artery disease (CAD) and microvascular dysfunction (MVD). The latter is usually defined by conditions of abnormal tone in the microvessels that regulate coronary resistance and match perfusion to metabolic demand [3,4]. The diagnosis of MVD often arises when stress cardiovascular imaging is abnormal in symptomatic individuals who lack significant obstructive epicardial CAD. Because of the critical role of the microcirculation in normal cardiac function, those with MVD have a long-term prognosis similar to that of patients with stable CAD [5,6].

In patients with obstructive CAD, blood metabolomic and lipidomic profiling has identified abnormal patterns that are either be a consequence of ischemic myocardial metabolism, or directly contribute to atherosclerosis [7,8,9]. Little is known about whether metabolic derangements exist in those with MVD. However, patients with chest pain but without obstructive CAD have been observed to have lower lysophosphatidyl choline [7]. The presence of overlap in “omic” profiles for CAD and MVD is reasonable to predict because (a) the two conditions have similar risk factors including hyperlipidemia, insulin resistance, and oxidative stress [10], and (b) both can produce periodic uncoupling of myocardial perfusion and metabolic demand. In this study we hypothesized that MVD would be associated with specific lipidomic profiles in a cohort of non-elderly patients with chest pain. The focus on lipidomics is based on the role of lipids and their metabolites in vascular cell and myocellular energetics and cell signaling. We also sought to determine whether any of these lipidomic patterns overlap with the fatty acid (FA), phospholipid, sphingolipid, and ceramide signatures associated with atherosclerotic CAD [8,11].

## 2. Results

**Figure 1:**
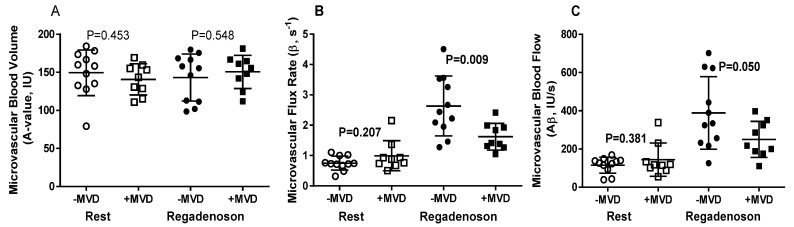
Contrast echocardiography data (mean ± STD) at rest and during regadenoson stress stratified according to presence of microvascular dysfunction (+MVD). Data include (**A**) microvascular blood volume, (**B**) microvascular flux rate, and (**C**) microvascular blood flow calculated by the product of microvascular blood volume and flux rate. Data from all subjects are shown.

A flow diagram of the study is provided in Figure S1. Nine patients (45%) had evidence of MVD, defined as delayed microvascular replenishment of contrast on MCE during vasodilator stress in at least one vascular territory. Quantitative analysis of myocardial perfusion on MCE showed no differences in resting MBV, microvascular flux rate (β), or microvascular blood flow between subjects classified as with versus without abnormal microvascular function (Figure 1). During regadenoson stress, those categorized as having MVD had significantly lower microvascular flux rate (β) and blood flow; thereby corroborating subjective classification of these patients (Figure 1). No significant differences in physiologic variables that determine oxygen demand, including heart rate and blood pressure, at rest or during stress were observed for subjects classified as having no abnormal microvascular function versus MVD (Table 1). Echocardiographic measurement of left ventricular geometry, cardiac output, and indices of myocardial work were also similar between groups (Table 2).

Clinical characteristics for the study subjects stratified according to microvascular function and non-obstructive CAD are shown in Table 3. There were no major demographic differences between those with normal versus abnormal microvascular function or with or without non-obstructive CAD. On average, two to three atherosclerotic risk factors were present in participants from both groups, and a large proportion of participants had a diagnosis of hyperlipidemia. There were no differences in the proportion of participants who had non-obstructive CAD versus completely normal coronary anatomy. Plasma lipid values measured on the day of the study showed average values that were normal or near-normal. Approximately one third of subjects in each group were treated with statins. Patients with MVD had higher HDL cholesterol (*p* = 0.04) and a trend toward higher total cholesterol (*p* = 0.06) when compared with those without abnormal microvascular function. When reclassified as patients with versus without non-obstructive CAD, there were no major differences between groups except for significantly higher use of cardiovascular medications, in particular antiplatelet agents and statins in those with non-obstructive CAD (Table 3).

**Figure 2:**
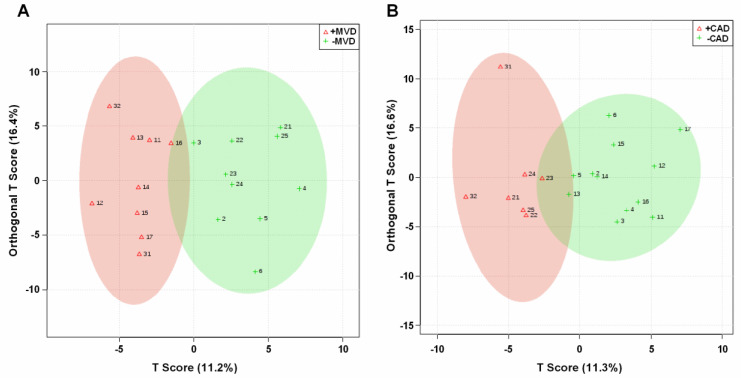
Partial least squares discriminant analysis (OPLS-DA) of plasma lipidomic profile discriminates between (**A**) participants with or without MVD and (**B**) participants with or without CAD. Signal intensities of 178 individual lipid species were normalized (quantile normalized), scaled (pareto scaling) and centered and then analyzed using OPLS-DA. The ellipses are drawn at 95% CI of normal distribution for a given group. Loading plots illustrate the proportion of variation accounted for by each component. Each number stands for one participant (1 to 6 for -MVD/-CAD, 11 to 17 for +MVD/-CAD, 21 to 25 for -MVD/+CAD, and 31 to 32 for +MVD/+CAD).

In the lipidomic analysis, a total of 178 lipid species were categorized in 15 lipid classes, of which the 13 lipid classes with >1 lipid species are shown in Table 4. MVD and non-obstructive CAD showed distinct differences in the plasma lipidomic profile, as visualized using orthogonal projections to latent structures discriminate analysis (OPLS-DA) (Figure 2). Subjects with MVD had lower plasma levels of triacylglycerol (TAG), the second most abundant lipid class, and tended to have higher levels of phosphatidyl ethanolamine, one of the minor lipid classes (Table 4). Plasma levels of other lipid classes did not differ between participants with versus those without MVD. In contrast, participants with non-obstructive CAD tended to higher plasma levels of TAG. Furthermore, lower levels of phosphatidyl ethanolamine. There were also trends towards lower levels of phosphatidyl choline plasmalogens and sphingomyelin.

**Figure 3:**
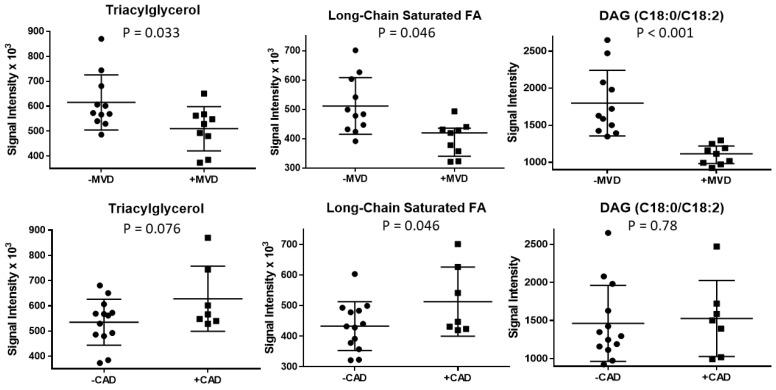
Abundance of triacylglycerol, long-chain saturated fatty acids, and diacylglycerol containing stearic and linoleic acid (DAG C18:0/C18:2) in plasma of participants stratified according to presence or absence of microvascular dysfunction (MVD) (**top** panels), or according to presence of non-obstructive coronary artery disease (CAD) (**bottom** panels). Data from all subjects are shown.

Among acylglycerols (comprehensively listed in Table 5), patients with MVD had lower values of acylglycerols containing long-chain saturated FA (Figure 3), specifically stearic acid (C18:0), heneicosanic acid (C21:0), and tricosanoic acid (C23:0); and long-chain unsaturated FA, specifically linoleic acid (C18:2) and eicosenoic acid (C20:1). In contrast, patients with non-obstructive CAD tended to have higher values of long-chain saturated FA. In addition, participants with MVD had higher delta 9 desaturation ratios for C16 and C18, indicative of higher hepatic desaturase activity.

Of the 178 putatively identified lipid species, seven lipid species fulfilled the statistical cut-off of *p* < 0.01 using the Kruskal–Wallis rank test for MVD classification (Table 6). All seven lipid species were DAG or TAGs and contained at least one C18 FA. The lipid species with the greatest discriminating power was a DAG with stearic acid (C18:0) and linoleic acid (C18:2), which correctly classified all participants (area under the receiver operating characteristic curve = 1) (Figure 3). None of these lipid species discriminated subjects with versus without non-obstructive CAD.

## 3. Discussion

MVD is a condition with many different clinical manifestations and a varied pathobiology. For many patients, MVD reflects an imbalance of vasoconstrictor and vasodilator processes that govern microvascular resistance under different physiologic states, commonly resulting in reduced peak hyperemic flow response [3,4]. Although functional abnormalities of the microcirculation are thought to be primary in the pathophysiology of MVD, some studies have revealed that abnormal coronary flow reserve can occur not from reduced hyperemic perfusion but from higher resting perfusion (the denominator in flow reserve calculation) [12], without a clear reason when considering classic determinants of myocardial oxygen demand or blood oxygen carrying capacity. This latter pattern could reflect an abnormality in cardiomyocyte metabolism since any conditions that impair generation of high energy phosphates would stimulate higher levels of perfusion for any given level of myocardial work [13].

In this study, we focused on lipidomic profiles in a cohort of non-elderly subjects that were deemed to have MVD on the basis of abnormal rest-vasodilator MCE perfusion imaging studies, but had no evidence of significant obstructive CAD on CTA. Evaluating “omic” profiles in this population was based on the need for biomarkers for risk assessment or for new pathway-specific therapies, and also a need to better understand disease pathobiology and its consequences.

In patients with obstructive CAD, abnormalities in metabolomic and lipidomic patterns have been characterized [7,8,9]. It is not entirely clear how many of these patterns are reflective of a process that contribute to vascular disease or instead are a result of periodic myocardial ischemia that can alter both metabolic and lipidomic signatures [14,15]. Because CAD and MVD share some of the same risk factors as well as some of the same consequences in terms of reduced flow response to demand, our initial step was to assess non-obstructive CAD and its risk factors in those with MVD. Those with MVD versus those without MVD were similar in terms of the prevalence of non-obstructive CAD with high-features on CTA. They were also similar with regards to the proportion of patients with traditional clinical or plasma lipid risk factors with the exception of slightly higher HDL cholesterol.

Our main novel finding was that MVD was linked to unique plasma lipidomic features. A single unique DAG containing stearic and linoleic acid classified all participants correctly according to their microvascular functional status. We identified several other DAG and TAG of similar chain length that also had very good classification properties, which makes it unlikely that the identified DAG is a false positive chance finding. A second major finding was that plasma lipidomic patterns that distinguished those with versus without MVD were distinct from those that distinguished those with versus without non-obstructive CAD that had at least one high-risk plaque feature on CT angiography. Moreover, the lipidomic patterns in MVD differed from the lipidomic changes in phospholipids (e.g., lysoPC), sphingomyelins, or ceramides described in larger trials of human subjects with CAD [7,16]. Instead, we observed differences in the lipid species of TAG and DAG, the FA of which are energy sources for mitochondrial FA oxidation for myocytes and myocardial cell populations including vascular cells. Specifically, lower long-chain FA were found in individuals with MVD. While these patterns have not been described in humans with CAD, it is worth noting that similar TAG patterns have been detected in mice after initiating an atherogenic diet [17].

The lipidomic findings in our study suggests a link between MVD and abnormal mitochondrial FA oxidation. The shift from long-chain FA to those primarily derived from incomplete FA oxidation could be indicative of either primary mitochondrial dysfunction or periodic ischemic effects rendering cardiomyocytes unable to generate sufficient ATP to satisfy demands. Mitochondrial dysfunction in the cardiomyocyte has been associated with reduced flow reserve primarily owing to a higher-than-expected basal (resting) flow through mechanisms that match perfusion to metabolic demand [18]. However, this explanations is insufficient alone to explain our results because MVD was defined based on abnormalities in peak hyperemic flow rather than on abnormal flow reserve (which can occur secondary to increased basal flow). Quantitative perfusion on MCE at rest was similar between groups.

Mitochondrial dysfunction and reduced oxidative metabolism is known to occur during myocardial ischemia, with an increase in plasma FA [19,20]. Ischemia leads to a preferential use of short-chain FA for mitochondrial FA oxidation [21] and a shift in energy fuel use toward glucose, amino acids, and ketones [2]. Accordingly, it is possible that the lipidomic findings in subjects with MVD are a consequence of repetitive ischemia, possibly as a compensatory response to allow the heart to adapt to periodic ischemia. It is also possible that the lipidomic patterns that we have identified are indicative of a pathophysiologic cause rather than effect of MVD. This idea is supported by the knowledge that certain anti-anginal medications including trimetazidine and ranolazine, the latter of which is one of only a handful of effective therapies effective in MVD [22] are known to inhibit FA oxidation.

There are important limitations to the current study. The study cohort was small and, thus, replication in a larger independent cohort is required; however, this is, to our knowledge, the first study, which examined lipidomic patterns in patients with MVD, indicating the transformational potential of this study. The final cohort size was influenced by the rigorous methods used to define our study population (patients had to be 35 to 55 years old with mild cardio vascular disease) to minimize the impact of comorbidities on lipidomic features. Independent follow-up studies are warranted to evaluate our lipidomic pattern in patients that are older, have diabetes, a history of cardio-vascular disease, or other geographical cohorts. MCE microvascular testing was performed with regadenoson rather than with other vasodilator agents that are endothelial-dependent. We selected regadenoson based on the notion that vascular response to an adenosine A2a-receptor agonist still requires the additional shear-mediated endothelial responses for achieving a full effect [23]. Classification of MVD and CAD was done subjectively by an expert, who was blinded to all other data, to ensure consistency of classification; however, this may impact replication. Given the small, observational nature of the study, participants were not matched based on demographic, risk factor, and medication differences, which may impact our results; it cannot be excluded that some of the medications may have impacted MVD classification and lipidomic patterns, warranting further studies. We do not think the modest differences in HDL found in our study were causative based on previous studies showing only a weak influence of HDL on coronary microvascular function and in the opposite pattern, better coronary flow reserve with higher HDL, than was found in our study [24].

In summary, lipidomic patterns linked to MVD and non-obstructive CAD differed markedly. The lipidomic pattern of patients with MVD was characterized by low abundance of long-chain saturated FA in acylglycerols with DAGs containing stearic and linoleic acid classifying all participants correctly. Long-chain saturated FA in acylglycerols serve as a nutritional source for myocardial energy expenditure, which may impact vasodilation and vasoconstriction. In contrast, patients with non-obstructive CAD had high abundance of long-chain saturated FA in plasma, indicating a tale of too little and too much of long-chain saturated FA in patients with MVD and non-obstructive CAD, respectively, justifying further investigation into the metabolic causes for these patterns.

## 4. Materials and Methods

### 4.1. Study Subjects

The study was approved by the Investigational Review Board at Oregon Health & Sciences University and registered with ClinicalTrials.gov (NCT02465554). A flow diagram of the study is provided in Supplemental Figure S1. Subjects 35 to 55 years of age who had been referred for CTA for evaluation of symptoms were prospectively screened for study eligibility over a two-year period. Subjects were excluded for history of CAD or other atherosclerotic disease, valvular heart disease (moderate or more), congenital heart disease, heart failure, presence of a myocardial bridge, vasculitis, pregnancy, contra-indications to regadenoson, or allergy to ultrasound contrast agents. Patients were considered eligible for the study if they had either (i) absence of non-obstructive CAD, defined as lack of detectable coronary plaque or stenosis on CTA and a coronary artery calcium score (CACS) of 0 (-CAD); or (ii) evidence of non-obstructive (<50% stenosis) coronary plaque in at least one coronary artery and at least one high-risk plaque feature defined below (+CAD). The requirement for at least one high risk feature was intended to provide power to determine whether strongly atherogenic “omic” profiles overlapped with those for MVD. The CTA studies were reviewed by an expert blinded to all other data to ensure eligibility. Consenting eligible patients underwent testing for MVD by vasodilator stress myocardial contrast echocardiography (MCE) microvascular perfusion imaging. Presence of microvascular dysfunction (+MVD) and absence of microvascular dysfunction (-MVD) defined below were classified by an expert [25]. All MCE studies were performed within 6 months of the CTA. Blood for fasting plasma lipid levels and lipidomics was drawn on the day of the MCE study. Patient history and active medication list reflected status at the time of the MCE study; medications were not adjusted by study personnel.

### 4.2. Coronary CT Imaging

Coronary CTA images were acquired using prospectively ECG-triggered protocol on a 256-multidetector-row scanner (Philips iCT, Cleveland, OH, USA) with the injection of iodinated contrast agent (70 mL of Omnipaque 350 mgI/mL). The imaging parameters included a tube current of 150 mAs, tube potential of 120 kVp, and gantry rotation of 280 ms. Images were reconstructed with a slice thickness of 0.8 mm and an increment of 0.4 mm. Coronary CTA images were analyzed on a workstation (Philips, IntelliSpace Portal 9.0, Cleveland, OH, USA) for the presence of coronary stenosis and plaque in all four major epicardial coronary arteries inclusive of the left main, according to the guidelines of the Society of Cardiovascular Computed Tomography. The total amount of coronary calcium was quantified using an Agatston score on non-contrast CT scan [26]. High risk features were defined by positive remodeling (remodeling index > 1.1 assessed in multiplanar reformatted images reconstructed in long axis and short axis view of the vessel), low CT attenuation plaque (presence of areas of low CT attenuation in non-calcified plaque with mean CT number in three regions of interest < 30 HU), napkin-ring sign (a ring-like peripheral higher attenuation of the non-calcified portion of the coronary plaque), and spotty calcium (calcified plaque with a diameter < 3 mm in any direction, length of the calcium less than 1.5 times the vessel diameter and width of the calcification < 2/3 of the vessel diameter) [27].

### 4.3. Microvascular Testing with Myocardial Contrast Echocardiography

All MCE and echocardiography studies were performed by a single expert sonographer. Patients were advised to abstain from caffeinated products for 48 h prior to the MCE study and blood draw. MCE perfusion imaging was performed with a contrast-specific multi-pulse algorithm (iE33, Philips Ultrasound, Andover, MA, USA) at a centerline frequency of 2.0 MHz. Imaging was performed at a mechanical index of 0.12–0.14 and gain was set at a level just below that which produced background myocardial speckle. Images were acquired in the apical 4-chamber, 2-chamber, and long-axis imaging planes. One vial (1.5 mL) of lipid-shelled octafluoropropane microbubbles (Definity, Lantheus Medical Imaging, North Billerica, MA, USA) was diluted to 30 mL total volume in normal saline and infused intravenously at 1.0 to 1.5 mL/min. A 5-frame high-power (mechanical index > 0.9) sequence was used to destroy microbubbles in the imaging sector through inertial cavitation, after which end-systolic frames were acquired to assess contrast replenishment. MCE was performed at rest and during intravenous regadenoson (0.4 mg) vasodilator stress. Presence of microvascular dysfunction (+MVD), defined by global or segmental lack of complete transmural microvascular refill within 2 s during vasodilator stress [28], was classified by an expert.

For quantitative analysis of microvascular function, digital video clips were analyzed using validated software (iMCE, Narnar LLC, Portland, OR, USA). Data were averaged for the three major coronary vascular territories which involved drawing of a transmural region-of-interest, including when defects were isolated to the subendocardium. Region-of-interests were standardized according to guideline-based definitions for coronary artery perfusion territories. The first post-destructive frame was digitally subtracted from all subsequent frames and background-subtracted time-intensity data were fit to the function:

*y* = *A*(1 − e^−*βt*^), (1)

where *y* is signal intensity at time *t*, *A* is the plateau intensity reflecting relative microvascular blood volume (MBV), and *β* is the rate constant reflecting microvascular blood flux rate. These kinetic curves were automatically generated by the analysis program with the user providing only identification of the first post-systolic frame and inputting the heart rate needed for time-intensity curve generation. Microvascular blood flow was quantified by the product of MBV and *β* [29]. Flow reserve and β-reserve were calculated by the ratio of values obtained during regadenoson to those at rest.

### 4.4. Echocardiography

Echocardiography (iE33, Philips Ultrasound, Andover, MA, USA) was performed to assess chamber dimensions and left ventricular function according to guidelines published by the American Society of Echocardiography [30]. Left ventricular ejection fraction (LVEF) and volumes were calculated using the modified Simpson’s method; while LV mass was calculated using the linear formula. Stroke volume was calculated using the product of left ventricular outflow tract area and the time-velocity integral measured by pulsed-wave spectral Doppler. Myocardial work was calculated by the product of stroke volume index, heart rate, and end-systolic blood pressure (0.90 × systolic BP).

### 4.5. Plasma Lipid Measurements

Plasma lipids were measured with a chemistry analyzer (Hitachi 704, Roche Diagnostics, Basel, Switzerland) using commercial reagents for total cholesterol, triglycerides, and HDL cholesterol (Roche Diagnostics); and for Lipoprotein(a) (Medtest DX, Canton, MI, USA). LDL cholesterol was calculated using the Friedewald’s formula and VLDL cholesterol was calculated as Triglycerides/5.

### 4.6. Metabolite and Lipid Extractions

Lipids standards were purchased from Avanti Polar Lipids, Inc. (Alabaster, AL, USA). Metabolites were extracted from 100 µL of plasma by 1:4 dilution with pre-chilled extraction solvent (methanol/ethanol; 50/50, *v*/*v*). Each sample was vortexed for 5 s followed by centrifugation at 15,000 RPM for 13 min under 4 °C. The supernatant was transferred to a glass vial and stored at −80 °C for future MS analyses. For lipid extraction, 100 µL of plasma was added to 300 µL of pre-chilled isopropyl alcohol containing 0.1% (*w*/*v*) butylated hydroxytoluene followed by 1 µg/mL of lipid standards. The mixture was vortexed for 5 min, and placed on ice for 30 min. After centrifugation at 15,000 RPM for 15 min, the supernatants containing the lipids were transferred into new glass vials. The lipid extracts were stored at −80 °C before MS analyses.

### 4.7. LC-MS^E^-Based Lipidomic Analysis

Lipidomic assays were performed on a Synapt G2 HDMS system (Waters, Milford, MA, USA) coupled to an ACQUITY UPLC system (Waters). For each biological sample, three replicate analyses were performed. Samples were analyzed independently based on a random list generated in R with blanks and QCs randomly interspersed in the injection sequence. Samples were placed in an auto-sampler with temperature set at 6 °C. An ACQUITY HSS T3 C18 column (2.1 mm × 100 mm, 18 µm, 100 Å) was used for lipid separation. The mobile phase included solvent A: acetonitrile (ACN)/water (40/60, *v*/*v*) with10 mM NH_4_HCO_3_ and solvent B: isopropyl alcohol/ACN/water (85/10/5, *v*/*v*/*v*) with 10 mM NH_4_HCO_3_. The column was equilibrated with 60% solvent A for 1 min. The gradient program started with 40% phase B/60% solvent A, reached 100% solvent B within 10 min, followed by column rinsing with 100% solvent B and re-equilibration with 40% B for 3 min and 1 min, respectively. The flow rate was 0.4 mL/min.

Analytes eluting from the LC were ionized using an electrospray ionization source (ESI) before entering the Synapt G2 mass spectrometer. The capillary voltage and cone voltage were set at ±3 KV and ±35 V, respectively, for both positive and negative polarities. The mass spectrometer was operated in the MS^E^ mode (MassLynx, Version 4.1). Three independent functions were automatically created in this acquisition mode: function 1 for MS1/low energy data with transfer collision energy set at 4.0 eV; function 2 for MS2/higher energy data with collision energy ramping from 20 to 55 eV; and function 3 for the LockSpray reference scan, for which 2 ng/µL Leucine enkephalin in ACN/H_2_O (50/50, *v*/*v*) was introduced via an external pump with constant flow rate of 10 µL/min. High resolution MS spectra for both low-energy and high-energy data were collected covering a range from 50 to 1500 *m*/*z*.

### 4.8. LC-MS/MS Metabolomic Analysis

All metabolomic assays were performed on a SCIEX TripleToF 5600 (Concord, ON, Canada) coupled to a Shimadzu Nexera UHPLC (Kyoto, Japan). For each biological sample, three technical replicates were performed, and for each replicate 10 µL of metabolite extract was injected. Samples were analyzed independently based on a random list generated in R with blanks and QCs randomly interspersed in the injection sequence. Metabolites were separated on a Inertsil Phenyl-3 column (4.6 mm × 150 mm, 100 Å, 5 µm; GL Science) held at 50 °C with flow rate at 400 µL/min. Solvent A was 100% water containing 0.1% FA and solvent B was 100% methanol with 0.1% FA. The elution gradient was as follows: 0 min, 5% B; 1 min, 5% B; 11 min, 30% B; 23 min, 100% B; 35 min, 100% B; 37 min, 5% B; 47 min, 5% B. Metabolites eluting from the LC column were ionized by electrospray ionization (ESI) using a Turbo V ion source operated at 5.5 KV and a source temperature of 550 °C. Ion source gas 1, gas 2 and curtain gas (all nitrogen) were set at 40, 50, and 25 respectively. The Sciex TripleTOF mass spectrometer was operated in the information dependent acquisition (IDA) mode. Collision energy was set at 40 V. The time-of-flight MS1 acquisition time was set to 0.25 s and MS/MS acquisition time to 0.17 s. The scan range was *m*/*z* 50–1250 for both MS1 and MS/MS. Two-minute auto-calibrations were performed after every third injection.

### 4.9. Lipidomic and Metabolomic Data Processing and Structure Assignment

MS raw data were processed by Progenesis QI (v2.2, Nonlinear Dynamics, Newcastle, UK), including feature detection, retention time alignment, data deconvolution and peak integration. Potential adducts were added, including [M+H]^+^, [M+Na]^+^, [M+NH_4_]^+^, and [M+H-H_2_O]^+^ for positive polarity; [M-H]^−^, and [M+FA-H]^−^ for negative polarity. Tentative structures were obtained by searching accurate masses (smaller than 10 ppm), mapping isotopic patterns (0.9) and tandem MS fragment patterns against public databases, such as LIPIDMAPS, HMDB, and METLIN. Manual tandem MS annotation further narrowed down tentative structures based on the reported fragmentation pathways and empirical retention times on the LC.

### 4.10. Statistical Methods

Imaging and lipidomic data were statistically analyzed using SAS version 9.2 (version 5.0, SAS Ins. Inc., Cary, NC, USA). To visualize differences in plasma standard lipid profile and plasma lipidomic profile between participants with or without MVD and with or without CAD, we used orthogonal partial least squares discriminant analysis (OPLS-DA) in MetaboAnalyst version 4.0 [30]. Concentrations of standard lipids and signal intensities of 178 individual lipid species were normalized (quantile normalized), scaled (pareto scaling) and centered and then analyzed using OPLS-DA. The ellipses are drawn at 95% CI of normal distribution for a given group. Loading plots illustrate the proportion of variation accounted for by each component.

Group-wise differences in proportions were assessed by Fisher’s exact test and are shown as *n* (%). Group-wise differences in continuous data that were normally distributed by Shapiro–Wilk test were assessed by Student’s *t*-test and are expressed as mean ± SD. For lipidomic data, a pooled variance estimate was used when variances were equal at *p* > 0.10 of a folded F-test and a Welch-Satterthwaite variance approximation was used when variance were unequal. Clinical and imaging data that were not normally distributed were assessed by a Mann–Whitney test, and data are shown as median and interquartile range (IQR). To determine statistical significance, we used for individual lipids and metabolites *p* ≤ 0.01 (the more stringent cut-off was used to account for multiple testing), for lipid groups, significance was declared at *p* < 0.05 and a tendency at 0.05 < *p* < 0.10. To determine individual lipid and metabolite species that best classify participants with or without MVD, area under the curve (AUC) values of receiver operating characteristic (ROC) curves were generated in Prism (version 6.0, GraphPad Software, San Diego, CA, USA). *p*-values for ROC-curves were obtained by a Kruskal–Wallis test.

## Figures and Tables

**Table 1:** Vital signs at rest and during vasodilator stress by presence of microvascular dysfunction (MVD).

	-MVD (*n* = 11)	+MVD (*n* = 9)	*p*-Value
**Rest**			
Heart rate (min^−1^)	68 ± 11	71 ± 14	0.45
Systolic BP (mm Hg)	118 ± 18	116 ± 17	0.82
Diastolic BP (mm Hg)	64 ± 10	66 ± 11	0.63
Double product (mm Hg/min)	7659 ± 2320	7989 ± 2628	0.78
**Regadenoson stress**			
Heart rate (min^−1^)	82 ± 19	79 ± 11	0.64
Systolic BP (mm Hg)	112 ± 20	119 ± 21	0.47
Diastolic BP (mm Hg)	64 ± 9	79 ± 11	0.26
Double product (mm Hg/min)	9219 ± 2625	9326 ± 1811	0.92

BP, blood pressure. Data are shown as mean ± STD; *p*-values for continuous data were obtained by a Student *t*-test.

**Table 2:** Left ventricular morphometry and function by presence of microvascular dysfunction (MVD).

	-MVD (*n* = 11)	+MVD (*n* = 9)	*p*-Value
LVIDd (cm)	4.67 ± 0.57	4.61 ± 0.73	0.84
LVIDs (cm)	3.01 ± 0.81	3.43 ± 0.76	0.30
IVSd (cm)	0.91 (IQR 0.67–0.99)	0.80 (IQR 0.58–1.15)	0.49
IVSs (cm)	1.36 (IQR 1.16–1.47)	1.10 (IQR 1.00–1.66)	0.37
PWd (cm)	1.36 ± 0.05	1.28 ± 0.12	0.11
Stroke volume (mL)	75.1 ± 12.4	74.8 ± 16.0	0.97
Cardiac output (L/min)	5.04 ± 0.97	5.20 ± 1.34	0.76
Stroke work (mL × mm Hg)	8818 ± 1942	8559 ± 1852	0.78
Myocardial work (×1000 mL × mm Hg/min)	596 ± 161	582 ± 199	0.87

LVIDd, diastolic left ventricular diameter; LVIDs, systolic left ventricular diameter; IVSd, diastolic interventricular septum thickness; IVSd, systolic interventricular septum thickness; PWd, diastolic posterior wall thickness. Data are shown as mean ± STD; *p*-values for continuous data were obtained by a Student *t*-test.

**Table 3:** Clinical characteristics and plasma lipid values stratified by presence of microvascular dysfunction (MVD) and non-obstructive CAD.

	-MVD (*n* = 11)	+MVD (*n* = 9)	*p*-Value	-CAD (*n* = 7)	+CAD (*n* = 13)	*p*-Value
Male, *n* (%)	5 (45%)	2 (22%)	0.37	3 (23%)	3 (43%)	0.61
Caucasian *n* (%)	11 (100%)	8 (89%)	1	12 (92%)	7 (100%)	1
Age (years)	49 ± 5	49 ± 5	0.88	49 ± 5	50 ± 6	0.60
BMI (kg/m^2^)	29.8 ± 6.9	27.2 ± 7.3	0.43	28.8 ± 7.6	28.7 ± 6.2	0.98
Obese, *n* (%)	5 (45%)	2 (22%)	0.37	5 (38%)	2 (29%)	0.37
Non-obstruct. CAD, *n* (%)	5 (45%)	2 (22%)	0.37			
MVD, *n* (%)				6 (46%)	2 (29%)	0.44
**Atherosclerotic risk factors (*n*)**	2.7 ± 1.1	2.8 ± 1.0	0.92	2.6 ± 0.8	3.0 ± 1.4	0.52
Any, *n* (%)	10 (91%)	8 (89%)	1	11 (92%)	6 (86%)	1
Family History, *n* (%)	6 (55%)	6 (67%)	0.67	7 (54%)	5 (71%)	0.64
Smoking history *n* (%)	2 (18%)	0 (0%)	0.49	2 (17%)	0 (0%)	0.51
Hypertension *n* (%)	6 (55%)	6 (67%)	0.67	7 (54%)	5 (71%)	0.64
Diabetes mellitus *n* (%)	0 (0%)	1 (11%)	0.45	0 (0%)	1 (11%)	0.35
Hyperlipidemia *n* (%)	9 (82%)	7 (78%)	1	8 (62%)	5 (71%)	1
**Medications, *n* (%)**	2.0 ± 0.8	2.1 ± 0.6	0.90	1.2 ± 0.9	3.6 ± 2.1	0.02
Any, *n* (%)	9 (82%)	7 (78%)	1	10 (77%)	6 (89%)	1
ACE-inhibitors, *n* (%)	1 (9%)	4 (44%)	0.13	3 (23%)	2 (29%)	1
Antiplatelets, *n* (%)	5 (45%)	1 (11%)	0.16	1 (8%)	5 (71%)	0.007
Beta Blockers, *n* (%)	3 (27%)	4 (44%)	0.64	3 (23%)	4 (57%)	0.17
CCBs, *n* (%)	2 (18%)	1 (11%)	1	1 (8%)	2 (29%)	0.27
Statins, *n* (%)	4 (36%)	3 (33%)	1	2 (15%)	5 (71%)	0.02
**Plasma Lipid Values**						
Total cholesterol (mg/dL)	178 ± 30	209 ± 13	0.06	201 ± 32	175 ± 40	0.07
LDL cholesterol (mg/dL)	100 ± 22	121 ± 31	0.10	115 ± 25	98 ± 31	0.12
HDL cholesterol (mg/dL)	56 ± 13	65 ± 11	0.04	67 ± 13	52 ± 11	0.13
VLDL cholesterol (mg/dL)	25 ± 7	24 ± 11	0.81	24 ± 9	24 ± 8	0.95
Triglycerides (mg/dL)	123 ± 36	118 ± 54	0.81	121 ± 47	120 ± 41	0.95
Lipoprotein(a) (mg/dL)	38 ± 48	13 ± 7	0.12	13 ± 10	51 ± 55	0.07

ACE, angiotensin converting enzyme; BMI, body mass index; CAD, non-obstructive coronary artery disease; CCB, calcium channel blocker; MVD, microvascular dysfunction. Continuous data are shown as mean ± STD and counts as *n* (%); *p*-values for continuous data were obtained by a Student *t*-test and for counts by Fisher’s exact test.

**Table 4:** Plasma lipid class levels of participants by microvascular dysfunction (MVD) and non-obstructive CAD *.

Lipid Class	Lipid Species	-MVD (*n* = 11)	+MVD (*n* = 9)	*p*-Value	-CAD (*n* = 13)	+CAD (*n* = 7)	*p*-Value
Total		1533 ± 129	1477 ± 112	0.32	1501 ± 107	1520 ± 155	0.74
Acylglycerols		636 ± 111	529 ± 90.4	0.03	555 ± 93	650 ± 129	0.07
Triacylglycerol	74	615 ± 110	510 ± 88.8	0.03	535 ± 91	628 ± 129	0.08
Diacylglycerol	15	18.1 ± 4.32	16.2 ± 4.07	0.34	16.5 ± 4.19	18.8 ± 4.11	0.26
Monoacylglycerol	3	3.26 ± 0.64	3.13 ± 0.84	0.68	3.16 ± 0.71	3.29 ± 0.79	0.71
Cholesteroyl ester	4	3.63 ± 0.65	3.90 ± 1.19	0.55	3.53 ± 0.82	4.15 ± 1.02	0.16
Glycerophospholipids		808 ± 108	851 ± 112	0.39	849 ± 114	786 ± 92	0.22
Lyso PC	6	11.6 ± 4.30	11.5 ± 3.14	0.93	11.6 ± 4.11	11.6 ± 3.19	0.99
Phosphatidyl choline	21	657 ± 87.2	694 ± 94.1	0.38	688 ± 96.3	647 ± 75.8	0.34
Phosphatidyl ethanolamine	9	7.61 ± 1.01	8.67 ± 1.42	0.07	8.45 ± 1.35	7.41 ± 0.90	0.08
Phosphatidyl serine	7	107 ± 20.7	114 ± 15.3	0.45	116 ± 16.3	100 ± 18.5	0.07
Plasmenyl PC	3	17.2 ± 7.48	16.3 ± 3.97	0.73	18.1 ± 7.00	14.5 ± 2.66	0.11
Plasmanyl PC	4	4.25 ± 1.57	5.00 ± 2.06	0.37	5.11 ± 1.93	3.63 ± 1.06	0.08
Plasmenyl PE	4	2.14 ± 0.94	1.85 ± 0.43	0.37	1.99 ± 0.77	2.04 ± 0.77	0.89
Sphingolipids		87.2 ± 16.6	94.2 ± 16.2	0.35	94.9 ± 13.6	82.0 ± 18.9	0.10
Ceramide	5	2.52 ± 1.94	1.56 ± 1.45	0.23	1.78 ± 1.41	2.66 ± 2.30	0.30
Sphingomyelin	21	84.7 ± 17.0	92.7 ± 16.8	0.31	93.1 ± 14.4	79.4 ± 18.8	0.08

* All values are sums of individual lipid species and expressed in peak intensity × 10^3^ and are shown as mean ± STD. *p*-values were obtained by a Student *t*-test. MVD, microvascular dysfunction. CAD, mild, non-obstructive coronary artery disease. Only classes only groups > 1 lipid species are shown. MVD, microvascular dysfunction; PC, phosphatidyl choline; PE, phosphatidyl ethanolamine.

**Table 5:** Plasma acylglycerol fatty acid levels of participants by microvascular dysfunction (MVD) and non-obstructive CAD *.

Fatty Acid	-MVD (*n* = 11)	+MVD (*n* = 9)	*p*-Value	-CAD (*n* = 13)	+CAD (*n* = 7)	*p*-Value
C12:0	2.57 ± 2.70	6.83 ± 6.60	0.10	4.66 ± 5.67	4.16 ± 4.57	0.84
C14:0	77.3 ± 32.2	88.7 ± 48.3	0.54	80.9 ± 37.6	85.3 ± 45.8	0.82
C14:1	5.33 ± 2.45	11.15 ± 9.47	0.11	8.64 ± 8.10	6.67 ± 4.84	0.57
C15:0	2.35 ± 1.19	2.55 ± 1.23	0.71	2.50 ± 12.3	23.4 ± 11.6	0.78
C16:0	349 ± 84.9	297 ± 51.9	0.13	303 ± 63.1	369 ± 80.7	0.06
C16:1	139 ± 26.0	126 ± 32.1	0.32	127 ± 30.5	144 ± 30.5	0.23
C17:0	3.71 ± 1.89	3.42 ± 1.63	0.72	3.36 ± 1.77	3981 ± 1.72	0.46
C17:1	11.0 ± 4.34	9.25 ± 2.47	0.29	10.1 ± 3.26	10,506 ± 4.56	0.81
C18:0	166 ± 40.82	107 ± 25.7	0.002	134 ± 45.9	150 ± 45.8	0.46
C18:1	452 ± 103	386 ± 68.6	0.12	395 ± 78.5	472 ± 104	0.08
C18:2	372 ± 72.9	294 ± 82.0	0.04	317 ± 72.5	372 ± 100	0.17
C18:3	44.4 ± 36.2	37.4 ± 17.6	0.31	77.8 ± 87.4	87.4 ± 30.4	0.48
C20:1	107 ± 23.4	81.4 ± 13.5	0.01	91.7 ± 19.8	102 ± 28.6	0.36
C20:3	5.16 ± 2.26	4.76 ± 3.59	0.76	3.91 ± 1.76	6.98 ± 3.54	0.06
C20:4	46.9 ± 12.3	39.5 ± 17.7	0.29	38.7 ± 12.0	52.6 ± 16.9	0.05
C21:0	8.41 ± 2.11	5.80 ± 2.22	0.02	6.96 ± 2.00	7.75 ± 3.33	0.52
C22:4	10.05 ± 4.31	7.54 ± 3.37	0.17	7.71 ± 3.22	11.18 ± 4.63	0.06
C22:6	7.71 ± 2.32	7.49 ± 4.21	0.88	6.47 ± 2.19	9.73 ± 3.87	0.07
C23:0	3.50 ± 1.30	1.66 ± 0.85	0.002	2.64 ± 1.49	2.71 ± 1047	0.92
MCSFA	82.3 ± 33.4	98.1 ± 54.6	0.43	88.1 ± 42.3	91.8 ± 49.7	0.86
LCSFA	546 ± 105	456 ± 61.1	0.04	474 ± 77.1	564 ± 109	0.05
FAΩ3	103 ± 27.3	71.6 ± 21.8	0.01	84.2 ± 28.1	97.1 ± 31.3	0.36
FAΩ6	434 ± 82.9	346 ± 97.5	0.04	368 ± 83.8	444 ± 110	0.10
FAΩ7/9	697 ± 138	592 ± 108	0.08	614 ± 111	717 ± 154	0.10
C22:6/18:3	0.09 ± 0.03	0.12 ± 0.06	0.14	0.09 ± 0.04	0.12 ± 0.05	0.22
C20:4/18:2	0.13 ± 0.03	0.13 ± 0.05	0.17	0.12 ± 0.03	0.14 ± 0.05	0.33
C16:1/0	0.87 ± 0.18	1.21 ± 0.39	0.03	1.01 ± 0.29	1.04 ± 0.43	0.85
C18:1/0	2.88 ± 1.00	3.81 ± 1.25	0.08	3.23 ± 1.09	3.42 ± 1.44	0.73

* All values are sums of individual lipid species and expressed in peak intensity × 10^3^ and are shown as mean ± STD. Only values with more >1000 peak intensity are shown. *p*-values were obtained using Student’s *t*-test. C, fatty acid carbon chain length; MCSFA, medium-chain saturated fatty acids; LCSFA, long-chain saturated fatty acid; FAΩ3, omega 3 fatty acids; FAΩ6, omega 6 fatty acids; FAΩ7/9, omega 7 and 9 fatty acids; C22:6/18:3, omega 3 elongation ratio; C20:4/18:2, omega 6 elongation ratio; C16:1/0, C16 delta 9 desaturation ratio; C18:1/0, delta 9 desaturation ratio.

**Table 6:** Best classifying plasma lipidomic species for microvascular dysfunction (MVD) *.

	-MVD (*n* = 11)	+MVD (*n* = 9)	*p*-Value	AUC of ROC-Curve (95% CI)
DAG (C18:0/C18:2)	1800 ± 443	1103 ± 131	0.0002	1.0
DAG (C18:1/C18:2)	1464 ± 377	859 ± 455	0.01	0.83 (0.63, 1)
TAG (C16:0/C18:1/C20:1)	15,119 ± 6336	9281 ± 2744	0.007	0.86 (0.70, 1)
TAG (C18:0/C18:0/C18:3)	54,822 ± 18,572	31,364 ± 9593	0.003	0.90 (0.74, 1)
TAG (C18:1/C18:1/C18:1)	158 ± 80	61 ± 46	0.004	0.88 (0.73, 1)
TAG (C18:1/C20:3/C23:0)	215 ± 182	67 ± 53	0.009	0.85 (0.38, 1)
TAG (C18:1/C20:4/C23:0)	1529 ± 636	674 ± 279	0.002	0.91 (0.78, 1)
TAG (C18:2/C20:4/C23:0)	1752 ± 604	916 ± 593	0.01	0.83 (0.63, 1)

* All values are expressed in peak intensity and are shown as mean ± STD. *p*-values for ROC-curves were obtained by a Kruskal–Wallis test; only plasma lipid species are shown that differ at *p* ≤ 0.01. C, fatty acid carbon chain length; 95% CI, 95% confidence interval; DAG, diacylglycerol; MVD, microvascular dysfunction; AUC of ROC-value, area under the curve of receiver operating characteristic; TAG, triacylglycerol.

## Data Availability

Lipidomic raw data are published on an open-access repository (Figshare.com, accessed on 1 September 2021).

## Supplementary Materials

The following are available online at https://www.mdpi.com/article/10.3390/metabo11100648/s1, Figure S1: Study enrollment flow chart. STROBE, Strengthening the Reporting of Observational Studies in Epidemiology. CACS, coronary artery calcium score; CAD, coronary artery disease; CVD, cardiovascular disease; MCE, myocardial contrast echocardiographye.
