# Supplementary material for: Plasma Lipidomic Patterns in Patients with Symptomatic Coronary Microvascular Dysfunction
Source: Metabolites. 2021 Sep 22;11(10):648. doi: 10.3390/metabo11100648 (PMC8540191; doi:10.3390/metabo11100648)
Supplement: Supplementary file 1 [file metabolites-11-00648-s001.zip › metabolites-1383331-supplementary.pdf]

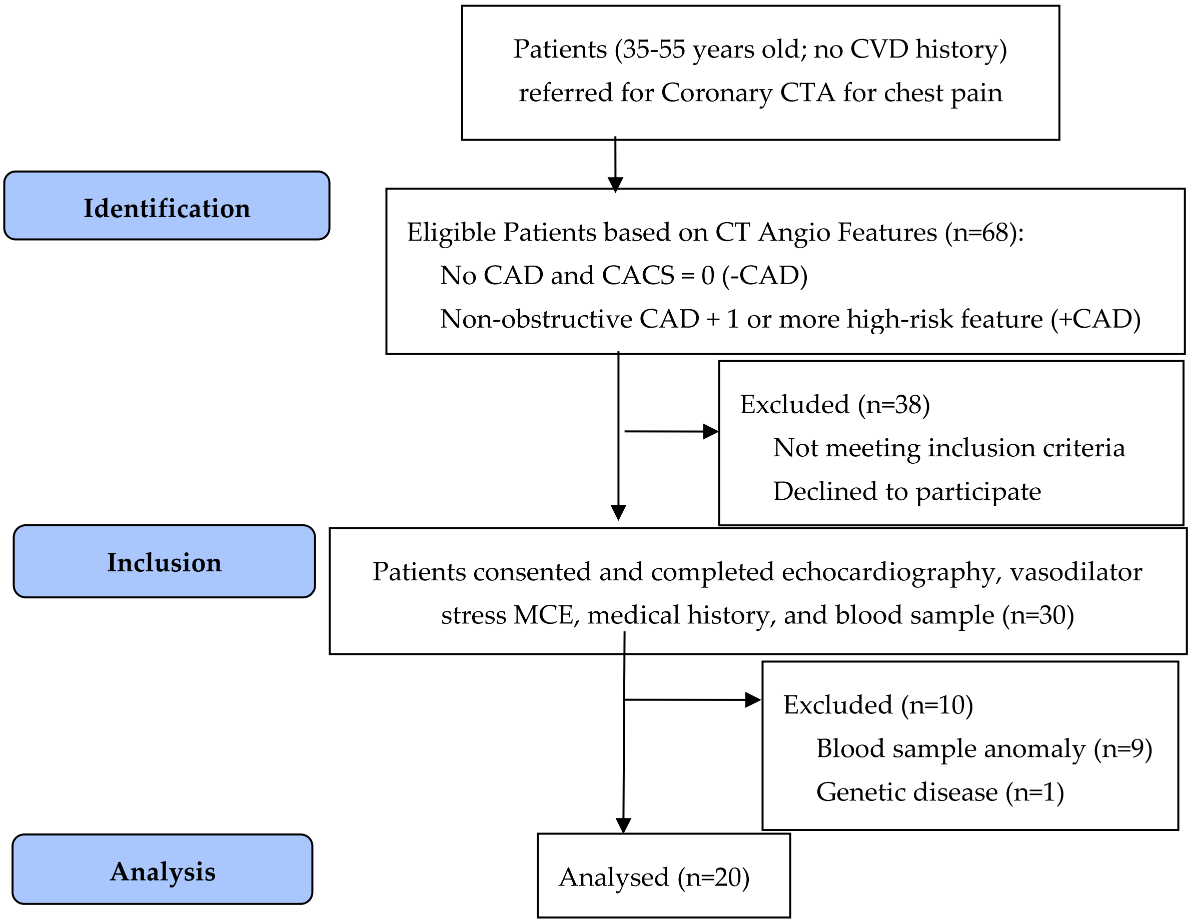

Supplemental Figure S1. Study enrollment flow chart. STROBE, Strengthening the Reporting of Observational Studies in Epidemiology. CACS, coronary artery calcium score; CAD, coronary artery disease; CVD, cardiovascular disease; MCE, myocardial contrast echocardiographye
